# Retrospective Analysis of Acute Kidney Injury in COVID-19 Infection: A Single-Center Study From Kerala

**DOI:** 10.7759/cureus.61772

**Published:** 2024-06-06

**Authors:** Sheela Mathew, Sreejith Ramaswamy, Shiji P V, Aquil Kalanad, Aaron G John

**Affiliations:** 1 Infectious Diseases, Government Medical College, Kozhikode, Kozhikode, IND; 2 Internal Medicine, Government Medical College, Kozhikode, Kozhikode, IND; 3 Gastrointestinal, Hepatopancreatobiliary, and Multi-Organ Transplant Surgery, Rajagiri Hospital, Aluva, IND

**Keywords:** covid-19, inflammatory markers, risk factors, acute kidney injury, mortality

## Abstract

Introduction

The COVID-19 pandemic, caused by SARS-CoV-2, has resulted in significant morbidity and mortality worldwide. While primarily a respiratory illness, COVID-19 can lead to multi-organ involvement, including acute kidney injury (AKI). This study aimed to retrospectively analyze the incidence, risk factors, and outcomes of AKI in COVID-19 patients.

Methods

A single-center retrospective study involving 232 severe COVID-19 patients requiring ICU admission was analyzed. Patients were categorized into two groups based on renal involvement: group A (with AKI or worsening of pre-existing chronic kidney disease) and group B (without renal injury). Data on demographics, comorbidities, clinical presentation, inflammatory markers, management strategies, and outcomes were collected and analyzed.

Results

AKI or worsening of pre-existing chronic renal disease was noted in 50.87% of cases, while the remaining 49.13% had severe COVID-19 pneumonia without renal injury. The mean age of patients in group A (with renal involvement) was higher compared to group B (without renal injury), with a significant male predominance observed in group A. AKI occurred within a short duration of fever, and cough was not a significant symptom. Comorbidities such as diabetes, hypertension, and chronic kidney disease were common in both groups, with hypertension significantly associated with AKI. Other significant comorbidities as risk factors for kidney injury included chronic liver disease, coronary artery disease, chronic kidney disease, and malignancy. Elevated inflammatory markers, including C-reactive protein (CRP), serum ferritin, and interleukin-6, were significantly associated with renal injury. There was no significant difference in the mortality rate between the two groups studied.

Conclusion

AKI or worsening of pre-existing kidney disease is a common event in severe COVID-19 infection. Patients, especially elderly males with comorbidities as mentioned, should be thoroughly monitored for worsening renal function, and steps like avoidance of nephrotoxic drugs and timely hemodynamic support may help avoid this dreaded complication to a certain extent and improve the prognosis in severe COVID-19 infection. Supportive care remains crucial in managing COVID-19 patients with renal involvement, emphasizing the need for the early detection and treatment of renal abnormalities. Long-term follow-up is essential to assess the impact of AKI on future kidney health.

## Introduction

COVID-19, a pandemic caused by SARS-CoV-2, a novel coronavirus, was first detected in Wuhan, China. By May 2023, it had affected more than 688 million cases worldwide, with over 6.8 million deaths. SARS-CoV-2 predominantly causes lung manifestations, ranging from mild symptoms to severe pneumonia and acute respiratory distress syndrome (ARDS). However, it often manifests as a multisystem disease with adverse outcomes, particularly impacting patients with pre-existing comorbidities such as diabetes, hypertension, obesity, and various kidney diseases [1].

Early reports from China noted hematuria and proteinuria but low rates of acute kidney injury (AKI) [2,3]. Subsequent reports from other countries revealed varying rates of AKI, with some series reporting up to 60% among seriously ill patients with poor prognosis [4].

The COVID-19 virus binds to the angiotensin-converting enzyme 2 (ACE2) receptor, primarily found in type II alveolar epithelial cells in the airways and lung parenchyma, but also present in many other organs including the kidneys, heart, brain, liver, vascular endothelium, and gastrointestinal tract [5]. As kidneys are the second most commonly affected organ after the lungs, patients with end-stage kidney disease (ESKD) are particularly vulnerable to severe COVID-19 due to their older age and high frequency of comorbidities such as diabetes and hypertension. The risk of in-hospital death due to COVID-19 was also higher among ESKD patients [6].

The host response to SARS-CoV-2 infection involves an initial specific adaptive immune response, which, if unsuccessful, can lead to a more severe and uncontrolled immune response characterized by a cytokine storm and vascular complications such as thrombosis. This cytokine storm, characterized by acute overproduction and uncontrolled release of proinflammatory mediators, along with dysregulation of the complement system, contributes to ARDS and hypercoagulability. The cytokine storm may promote apoptosis or necrosis of T cells and is commonly observed in patients with severe disease requiring intensive care unit (ICU) admission, as well as those who have elevated levels of IL-6, IL-1, IL-2, IL-7, IL-17, and TNF-α [7].

Dysregulation of the complement system and hypercoagulability, along with consumption of clotting factors and development of microvascular thrombi, contribute to the illness, as indicated by prolongation of prothrombin time and international normalized ratio (PT/INR) and partial thromboplastin time (PTT), along with significant increases in D-dimer levels and decreases in fibrinogen levels [8]. Elevated D-dimer levels have been observed as a significant predictor of mortality in several studies, leading to the inclusion of anticoagulation in the management protocol for COVID-19. A study conducted by Li et al. showed that D-dimer levels were elevated in 70% of the 182 patients analyzed and elevated levels of D-dimer were more common in severe disease [8].

AKI and worsening of pre-existing chronic kidney disease (CKD) were significant problems observed in COVID-19 patients, adding to management issues and overburdening healthcare systems during the pandemic. About one-third of COVID-19 patients exhibited renal involvement, with elevated blood urea and serum creatinine levels [9]. The incidence of AKI was particularly high in patients requiring intensive care treatment. Its severity was directly related to mortality, especially in those with high blood urea and serum creatinine levels at admission [10].

Older age and comorbidities such as CKD, cerebrovascular disease, and hypertension were associated with more severe lung involvement due to COVID-19. Mechanisms leading to kidney involvement include direct cytopathic damage by the virus, cytokine release syndrome, prerenal damage due to hypovolemia, acute tubular necrosis secondary to infection or rhabdomyolysis, and drug-induced nephrotoxicity [11].

Patients with CKD are predisposed to respiratory tract infections and pneumonia due to defects in their immune status and chronic inflammatory state. Various studies have observed a significant association of severe COVID-19 in patients with CKD. Post-renal transplant patients are also at high risk of COVID-19 due to their chronic immunosuppression, which makes them susceptible to bacterial and viral infections. The mortality rate in CKD patients on maintenance hemodialysis with COVID-19 was higher compared to the general population [12].

Our study aimed to assess the incidence of AKI or exacerbation of pre-existing CKD in patients with severe COVID-19 requiring ICU admission. Other objectives included comparing clinical characteristics and demographic profiles, evaluating comorbidity associations, analyzing inflammatory marker levels, assessing respiratory support impacts, determining treatment strategy effectiveness, investigating ICU stay duration and mortality rates, and identifying risk factors for mortality in COVID-19 patients with renal injury.

## Materials and methods

Patients with severe COVID-19 (category C), defined as individuals confirmed positive for COVID-19 by RT-PCR and exhibiting red flag signs such as breathlessness, chest pain, drowsiness, fall in blood pressure, hemoptysis, cyanosis, or experiencing worsening of underlying chronic conditions including lung, heart, liver, and kidney diseases, neurological disorders, hypertension, hematological disorders, uncontrolled diabetes, cancer, HIV/AIDS, and cardiovascular diseases, were selected for this study. These patients required ICU admission at Government Medical College, Kozhikode. Among them, patients displaying features of renal dysfunction, either as AKI or with worsening of pre-existing CKD, were designated as group A. They were compared with patients with severe COVID-19 but without renal injury, designated as group B. AKI was defined according to the Kidney Disease Improving Global Outcomes (KDIGO) criteria, which include a rise in serum creatinine levels more than 0.3 mg/dl or greater within 48 hours, a 50% increase in serum creatinine levels known or presumed to have occurred within the past seven days, or a decrease in urine output to less than 0.5 mL/kg/hour for more than six hours [13]. Patients meeting the above criteria were included in the study based on the sequential order of admission to the COVID-19 ICU from October 2021 to November 2022. Patients with pre-existing CKD without worsening, individuals with category A or B COVID-19 infection, those aged less than 18 years, and pregnant females were excluded from our analysis.

Data collected from medical records encompassed symptoms like fever, cough, and dyspnea, along with a history of renal disease, while investigations included inflammatory markers and serial renal function tests to rule out AKI, with treatment strategies involving antiviral drugs like remdesivir, anticoagulation, steroid therapy, and ventilation support options including face masks, high-flow nasal cannula, non-invasive ventilation, or invasive ventilation. The length of ICU stay and disease outcome were also studied. Furthermore, another subgrouping of expired and survived patients was made, and an analysis of individual factors related to mortality was also conducted. Data were entered into Microsoft Excel and analyzed using IBM SPSS Statistics for Windows, Version 24.0 (Released 2016; IBM Corp., Armonk, New York, United States).

Categorical data are presented as frequency and percentage. Continuous data are presented as mean +/- SD for normal distribution and median and quartiles for non-normally distributed data. Rate comparisons were performed by the chi-squared test, t-test, Wilcoxon rank-sum test, Wilcoxon signed-rank test, or Kruskal-Wallis test. Multivariate binary logistic regression was used to select and estimate the association between statistically significant variables in univariate analysis and renal injury (inclusion criteria p<0.05). Results were presented as odds ratio (OR) with a 95% confidence interval (95% CI) and p-value.

## Results

A total of 232 patients with severe COVID-19 who required ICU admission were selected for the study. Among them, 118 patients who exhibited features of AKI or worsening of pre-existing CKD (group A) were compared with 114 patients with severe COVID-19 without renal injury (group B). Group A consisted of 77 patients with AKI and 41 patients with pre-existing CKD admitted with worsening of symptoms.

The mean age of patients with COVID-19 and kidney involvement (group A) was 64.5±10.95, whereas the mean age of patients with COVID-19 and no kidney involvement (group B) was 58.6±15.6. In group A, 80.5% (n=95) were males, whereas in group B, 59.6% (n=68) were males. The male-to-female sex ratio was 80.5:19.5 in group A compared to 59.6:40.4 in group B.

Analyzing the symptoms of COVID-19, fever was present in 54.2% (n=64) of patients in group A, while fever was reported by 81.6% (n=93) in group B. Similarly, dyspnea was present in 80.5% (n=95) of group A compared to 92.1% (n=105) in group B. Cough was present in only 39% (n=46) of patients in group A, while it was significantly more common in group B with 80.7% (n=92) reporting it.

Analyzing comorbid illnesses, diabetes mellitus was the most common comorbidity in both groups (67% and 54% in groups A and B, respectively), followed by hypertension (60% and 36% in groups A and B, respectively) and coronary artery disease (37% and 13% in groups A and B, respectively). The prevalence of other comorbid illnesses in both groups is given in Table 1.

**Table 1:** Comparison between AKI group and non-AKI group AKI: acute kidney injury; CAD: coronary artery disease; CKD: chronic kidney disease; CLD: chronic liver disease; COPD: chronic obstructive pulmonary disease; CVA: cerebral vascular accident; DM: diabetes mellitus; HD: hemodialysis; HTN: hypertension; ICU: intensive care unit; NIV: non-invasive ventilation; NRBM: non-rebreather mask; NSAID: nonsteroidal anti-inflammatory drug

	AKI (n=118)	Non-AKI (n=114)	P-value
Age (in years)	64.52±10.95	58.60±15.63	.001
Sex males (%)	95 (80.5%)	68 (59.6%)	.001
Fever in days	1±4.5	2±3	.000
Dyspnea in days	2±2.5	1±1	.631
Cough in days	0±2.5	2±2	.000
History of CKD	40 (33.9%)	0	.000
History of DM	67 (56.8%)	54 (47.4%)	.15
History of HTN	60 (50.8%)	36 (31.6%)	.003
History of CVA	11 (9.3%)	10 (8.8%)	.884
History of malignancy	3 (2.5%)	11 (9.6%)	.023
History of CLD	6 (5.1%)	16 (14.0%)	.02
History of COPD	18 (15.3%)	12 (10.5%)	.283
History of CAD and cardiac disease	37 (31.6%)	13 (11.4%)	.000
History of any treatment regular/irregular	96 (81.4%)	64 (56.1%)	.000
History of documented hypotension	17 (14.4%)	10 (8.8%)	.181
Mode of respiratory support			.000
No respiratory support	0 (6.8%)		
Face mask	32 (27.1%)	53 (46.5%)	
NRBM	20 (16.9%)	5 (4.4%)	
NIV	32 (27.1%)	47 (41.2%)	
Mechanical ventilation	26 (22%)	9 (7.9%)	
History of NSAID use	2 (1.7%)	5 (4.4%)	.231
HD requirement	22 (18.6%)		
Tocilizumab	2 (1.7%)	13 (11.4%)	.003
Plasma	6 (5.1%)	3 (2.6%)	.33
Steroids	106 (89.8%)	109 (95.6%)	.091
Anticoagulation	96 (81.4%)	82 (71.9%)	.089
Length of ICU stay	4.7±2.7	6.8±5.9	.001
Mortality	56 (47.5%)	48 (42.1%)	.412

Seventeen patients (14.4%) in group A had a history of documented hypotension, while 10 patients (8.8%) in group B had the same. Only two patients (1.7%) in group A and five patients (4.4%) in group B reported consuming nonsteroidal anti-inflammatory drugs (NSAIDs) during the course of illness.

The mean values for blood urea on days 1, 3, and 5 of admission into the ICU (group A) were 88.4 mg/dL, 108.7 mg/dL, and 117.9 mg/dL, respectively. The mean values for serum creatinine on days 1, 3, and 5 of admission (group A) were 2.88 mg/dL, 3.16 mg/dL, and 3.4 mg/dL, respectively. The mean serum potassium values on days 1, 3, and 5 of admission (group A) were 4.65 mEq/L, 4.84 mEq/L, and 4.53 mEq/L, respectively (Table 2).

**Table 2:** Comparison of biochemical parameters in AKI group and non-AKI group AKI: acute kidney injury

	AKI (n=118)	Non-AKI (n=114)	P-value
Blood urea on day 1 (mg/dL)	88.42±48.65	29.13±8.54	.000
Blood urea on day 3 (mg/dL)	108.77±54.77	31.68±8.89	.000
Blood urea on day 5 (mg/dL)	117.92±63.88	30.14±8.19	.000
Serum creatinine on day 1 (mg/dL)	2.89±2.63	0.83±0.20	.000
Serum creatinine on day 3 (mg/dL)	3.16±2.39	0.82±0.15	.000
Serum creatinine on day 5 (mg/dL)	3.40±2.46	0.79±0.17	.000
Serum potassium on day 1 (mEq/L)	4.66±0.95	4.03±0.75	.000
Serum potassium on day 3 (mEq/L)	4.84±1.18	3.94±0.71	.000
Serum potassium on day 5 (mEq/L)	4.54±1.03	4.14±0.63	.011
Acidosis			0.001
No acidosis	59 (51.8%)	34 (28.8%)	
Mild acidosis (pH 7.3-7.35)	46 (39%)	25 (21.9%)	
Moderate acidosis (pH 7.2-7.3)	15 (12.7%)	11 (9.6%)	
Severe acidosis (pH <7.2)	8 (6.8%)	1 (0.9%)	
Alkalosis (pH >7.45)	15 (12.7%)	18 (15.8%)	

**Figure 1:**
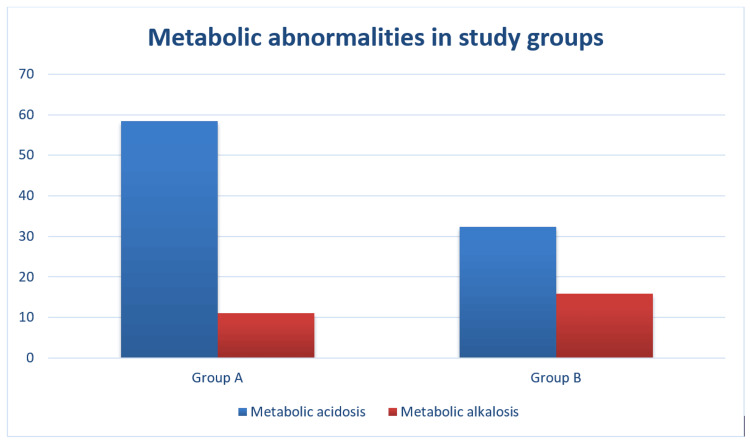
Metabolic abnormalities in group A and group B

Metabolic acidosis was present in 69 patients (58.5%), out of which eight patients (6.8%) had severe acidosis (defined as pH less than 7.2) in group A, while 37 patients (32.4%) had acidosis in group B, out of which only one patient (0.9%) had severe acidosis. A pH between 7.2 and 7.3 was classified as moderate acidosis and between 7.30 and 7.35 was classified as mild acidosis. Metabolic alkalosis (pH >7.45) was present in 15 patients (12.7%) in group A, while 18 patients (15.8%) had the same in group B (Figure 1).

Inflammatory markers were tested among both groups, including lactate dehydrogenase (LDH), D-dimer, creatine kinase MB (CK-MB), myoglobin, troponin I, B-type natriuretic peptide (BNP), C-reactive protein (CRP), serum ferritin, and interleukin-6 levels in as many patients as possible. Details are given in Table 3.

**Table 3:** Mean values of inflammatory markers in both groups BNP: B-type natriuretic peptide; CK-MB: creatine kinase MB; CRP: C-reactive protein; IL-6: interleukin-6; LDH: lactate dehydrogenase; Trop I: troponin I

Parameter	Group A		Group B		
	n	Mean value	n	Mean value	P-value
CK-MB (IU/L)	53	9.11±14.05	41	19.99±101.66	0.426
Myoglobin (ng/mL)	51	381.17±146.02	36	287.66±406.36	0.095
Trop I (ng/mL)	31	0.05±0.05	24	0.05±0.0	0.797
BNP (pg/mL)	65	183±706.4	29	50±288.3	0.028
Ferritin (ng/mL)	43	1274.00±1114.82	33	616.58±481.64	0.001
LDH (U/L)	33	651.3±420.2	43	753.2±477.8	0.335
CRP (mg/dL)	45	123.06±101.85	34	72.28±62.86	0.012
D-dimer (ng/mL)	68	2404.3±1928.4	70	1801.4±1635.5	0.069
IL-6 (pg/mL)	17	214.57±246.802	20	83.20±90.718	0.033

While evaluating the management strategies in both groups, an antiviral drug (remdesivir) was given in 36.4% (n=43) of group A patients and 64.9% (n=73) in group B patients. Steroids were given in 89.8% (n=106) of group A patients and 95.6% (n=109) in group B. Tocilizumab was given in 1.7% (n=2) of group A patients and 11.4% (n=13) of patients in group B. Anticoagulation was given in 81.4% (n=96) of group A patients and 71.9% (n=82) of group B patients. Hemodialysis was done as part of treatment in 22 patients in group A (18.6%), while hemodialysis was not done in any patients in group B as there was no renal injury among them. The mean duration of ICU stay in group A was 4.7 days, while in group B it was 6.8 days.

**Figure 2:**
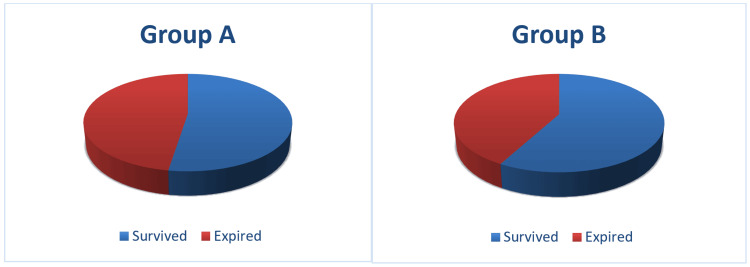
Mortality in group A and group B

A total of 104 patients (44.8%) expired in the study population. Out of the total 118 patients in group A, both acute and worsening of pre-existing CKD requiring ICU admission, 56 patients (47.5%) expired. In group B, 48 patients (42.1%) expired (p=0.412) (Figure 2).

All surviving patients were advised a follow-up at the community level.

Multivariate binary logistic regression analysis was done to select and estimate the association between statistically significant risk factors in univariate analysis and renal injury (Table 4).

**Table 4:** Results of multivariate binary logistic regression analysis AKI: acute kidney injury; BNP: B-type natriuretic peptide; CAD: coronary artery disease; CKD: chronic kidney disease; CK-MB: creatine kinase MB; CLD: chronic liver disease; COPD: chronic obstructive pulmonary disease; CRP: C-reactive protein; CVA: cerebral vascular accident; DM: diabetes mellitus; HD: hemodialysis; HTN: hypertension; ICU: intensive care unit; IL-6: interleukin-6; LDH: lactate dehydrogenase; NIV: non-invasive ventilation; NRBM: non-rebreather mask; Trop I: troponin I

	Non-AKI (n= 114)	AKI (n=118)	P-value	Multivariate p-value	OR with 95% CI
Sex males (%)	68 (59.6%)	95 (80.5%)	0.001	0.092	
History of CKD	0	40 (33.9%)	0.000	0.995	
History of renal transplant	15 (13.2%)	4 (3.4%)	0.007	0.999	
History of DM	54 (47.4%)	67 (56.8%)	0.15	0.706	
History of HTN	36 (31.6%)	60 (50.8%)	0.003	0.0739	
History of CVA	10 (8.8%)	11 (9.3%)	0.884	0.170	
History of malignancy	11 (9.6%)	3 (2.5%)	0.023	0.036	7.4 (1.127-48.180)
History of CLD	16 (14.0%)	6 (5.1%)	0.02	0.483	
History of COPD	12 (10.5%)	18 (15.3%)	0.283	0.768	
History of CAD and cardiac disease	13 (11.4%)	37 (31.6%)	0.000	0.233	
History of any treatment regular/irregular	64 (56.1%)	96 (81.4%)	0.000	0.034	0.316 (0.109-0.915)
History of documented hypotension	10 (8.8%)	17 (14.4%)	0.181	0.439	
Mode of respiratory support			0.000	0.007	
No respiratory support		0 (6.8%)			
Face mask	53 (46.5%)	32 (27.1%)		0.999	
NRBM	5 (4.4%)	20 (16.9%)		0.003	0.103 (0.023-0.450)
NIV	47 (41.2%)	32 (27.1%)		0.640	
Mechanical ventilation	9 (7.9%)	26 (22%)		0.030	0.228 (0.060-0.865)
History of NSAID use	5 (4.4%)	2 (1.7%)	0.231	0.997	
HD requirement		22 (18.6%)		0.998	
Acidosis			0.001	0.416	
No acidosis	59 (51.8%)	34 (28.8%)			
Mild acidosis (pH 7.3-7.35)	25 (21.9%)	46 (39%)			
Moderate acidosis (pH 7.2-7.3)	11 (9.6%)	15 (12.7%)			
Severe acidosis (pH <7.2)	1 (0.9%)	8 (6.8%)			
Alkalosis (pH >7.45)	18 (15.8%)	15 (12.7%)			
Tocilizumab	13 (11.4%)	2 (1.7%)	0.003		
Plasma	3 (2.6%)	6 (5.1%)	0.33		
Steroids	109 (95.6%)	106 (89.8%)	0.091	0.253	
Anticoagulation	82 (71.9%)	96 (81.4%)	0.089	0.048	0.322 (0.104-0.990)
Age (in years)	58.60±15.63	64.52±10.95	0.001	0.017	1.047 (1.008-1.088)
Fever (in days)	2±3	1±4.5	0.000	0.024	0.771 (0.616-0.966)
Dyspnea (in days)	1±1	2±2.5	0.631	0.919	
Cough (in days)	2±2	0±2.5	0.000	0.740	
CK-MB (IU/L)	19.99±101.66	9.11±14.05	0.426		
Myoglobin (ng/mL)	287.66±406.36	381.17±146.02	0.095		
Trop I (ng/mL)	0.05±0.0	0.05±0.05	0.797		
BNP (pg/mL)	50±288.3	183±706.4	0.028		
Ferritin (ng/mL)	616.58±481.64	1274.00±1114.82	0.001		
Blood urea on day 1 (mg/dL)	29.13±8.54	88.42±48.65	0.000		
Blood urea on day 3 (mg/dL)	31.68±8.89	108.77±54.77	0.000		
Blood urea on day 5 (mg/dL)	30.14±8.19	117.92±63.88	0.000		
Serum creatinine on day 1 (mg/dL)	0.83±0.20	2.89±2.63	0.000		
Serum creatinine on day 3 (mg/dL)	0.82±0.15	3.16±2.39	0.000		
Serum creatinine on day 5 (mg/dL)	0.79±0.17	3.40±2.46	0.000		
Serum potassium on day 1 (mEq/L)	4.03±0.75	4.66±0.95	0.000		
Serum potassium on day 3 (mEq/L)	3.94±0.71	4.84±1.18	0.000		
Serum potassium on day 5 (mEq/L)	4.14±0.63	4.54±1.03	0.011		
Length of ICU stay (days)	6.8±5.9	4.7±2.7	0.001	0.074	

Older age (p=0.017, OR with 95% CI: 1.047 (1.008-1.088)), short duration of fever (p=0.024, OR with 95% CI: 0.771 (0.616-0.966)), past history of irregular treatment for chronic diseases (p=0.034, OR with 95% CI: 0.316 (0.109-0.915)), history of malignancy (p-value=0.036, OR with 95% CI: 7.4 (1.127-48.180)), use of NRBM (p=0.003, OR with 95% CI: 1.047 (0.103-0.450)), and mechanical ventilation (p=0.030, OR with 95% CI: 0.228 (0.060-0.865)) were found to be significant factors predicting nephrotoxicity.

## Discussion

It's a well-established fact that increased age is a strong risk factor for COVID-19 severity [14]. Even after adjustment for other age-related risk factors, advanced age remains a significant risk for disease severity. The mean age in both groups (group A (COVID-19 with kidney involvement) and group B (COVID-19 without kidney involvement)) showed a statistically significant higher value in group A (p=0.001). This suggests that old age is a significant risk factor for developing renal injury in COVID-19. Lin et al., in a meta-analysis regarding risk factors for AKI and prognosis in COVID-19, observed that age over 60 years and severe COVID-19 were independent risk factors for AKI with OR 3.53, 95% CI (2.92-4.25) and OR 6.07, 95% CI (2.53-14.58), respectively [15].

Similar to age, male sex was observed to be affected more and with more severity. This increased susceptibility and vulnerability in males may be due to various reasons. Emerging evidence suggests that ACE2, a co-receptor for SARS-CoV-2 viral entry, has higher expression in males [16]. The male-to-female ratio was higher in group A, and it was statistically significant, indicating male sex as a significant risk factor for renal injury.

Analyzing the symptoms of COVID-19, fever was mainly present in group B (81.6%) compared to 54.2% in group A. This suggests that AKI is associated with short-duration fever, which was statistically significant (p=0.002). Similarly, cough was present in only 39% of patients in group A, while it was significantly more common in group B (80.7%), which was also statistically significant (p=0.000). Compared to the above symptoms, dyspnea was present in both groups without any statistical significance. This means that fever and cough were less common in patients with COVID-19 and renal injury compared to those without renal injury.

Analyzing comorbid illnesses, diabetes was the most common comorbidity observed, even though not significant, followed by hypertension. Those with hypertension were more vulnerable to AKI (p=0.003). Other significant comorbidities as risk factors for kidney injury included chronic liver disease (p=0.02), coronary artery disease (p=0.000), CKD (p=0.000), irregular treatment for chronic diseases (p=0.000), and malignancy (p=0.023). Renal functions have an impact on the heart, lungs, and other systems. Many patients with severe COVID-19 have co-existing chronic conditions like hypertension, diabetes, COPD, and liver disease. Such co-existing diseases increase kidney disease. Organ systems like the heart, lungs, liver, and kidneys rely on and support each other's functions, so when the new coronavirus causes damage in one area, others might be at risk. That may be why kidney damage arising in patients with COVID-19 is a possible warning sign of a serious, even fatal course of the disease. In the early reports of COVID-19 from China, patients with cancer made up a small proportion (0.9%) of COVID-19 cases but had more severe presentations (30% versus 16%) and higher mortality (5.6% versus 2.3%) than the general population [17]. The presence of documented hypotension and a history of NSAID usage during the course of illness did not show any statistical significance either in developing renal injury or mortality.

Inflammation is a key player in the pathogenesis of AKI. SARS-CoV-2 infection may trigger the activation of multiple inflammatory pathways like cytokine storm, CRP, complement activation, ACE2, and lung-kidney crosstalk to cause AKI and other organ involvement [18]. When various inflammatory markers were analyzed, the values in both groups were significantly elevated from baseline. CRP, serum ferritin, and IL-6 showed statistically significant differences between the two study groups, indicating a specific role in AKI mediation. IL-6 is a mediator as well as a biomarker of AKI and a predictor of AKI in different diseases like cardiovascular disease, liver disease, and renal disease [19]. Higher levels correlate with severity, the need for mechanical ventilation, and in-hospital mortality in COVID-19 infection [20]. CRP, produced by the liver and many inflammatory cells, is an acute-phase protein. It has been widely used in clinical settings as an acute inflammation biomarker. Serum CRP level is usually elevated in COVID-19 infection, and the level often predicts the clinical outcome. Also, COVID-19 patients with AKI show higher levels of CRP than those without AKI [21]. However, the pathogenic role and mechanisms of CRP in COVID-19-associated AKI remain largely unknown.

Ferritin, an important mediator of immune dysregulation with a proinflammatory effect and immune suppressive effect, substantially contributes to the cytokine storm. The release of proinflammatory cytokines, cellular damage, metabolic acidosis, reactive oxygen species generation, and secondary tissue damage are responsible for high ferritin levels in association with COVID-19. Vargas-Vargas and Cortés-Rojo report that the level of ferritin is high in severe COVID-19 and very severe COVID-19, but the level in very severe COVID-19 is significantly higher [22]. In the present study, ferritin level was very high in both groups (1274.00±1114.82 in group A versus 616.58±481.64 in group B) but was significantly higher in group A (p=0.001). Similar observations were made by Mohammad et al., who reported that serum ferritin levels were higher in patients with COVID-19-related AKI (median 1016 ng/mL (IQR 516-2534)) compared to those without AKI (median 680 (IQR 315-1416)) [23]. Similar findings are also reported by Hansrivijit et al. [24]. This observation was further confirmed by a systematic review, meta-analysis, and meta-regression analysis by Kaushal et al. [25].

Other inflammatory markers analyzed like D-dimer, BNP, LDH, CK-MB, troponin I, and myoglobin levels were markedly elevated in both groups but did not show any statistical difference between the two groups. This means that among biomarkers, elevated levels of CRP, serum ferritin, and IL-6 were significant in patients with COVID-19 and renal injury compared to patients without renal involvement. Acidosis was present in both groups and significantly higher in group A (p=0.001). Both renal injury and sepsis can cause acidosis; however, moderate to severe metabolic acidosis was found to have statistical significance in relation to the development of renal injury and mortality (p-=0.000). Assessing the mortality rate, there was no statistically significant mortality difference between the two groups. Ninety-three percent in group A and all patients in group B received some sort of respiratory support. Those with mechanical ventilation had a higher chance of renal injury (p=0.000). The duration of ICU stay was longer in the non-AKI group (p=0.001).

Our study was a single-center study conducted in a single ICU and was retrospective in design, which may limit the generalizability of the findings to other settings with different patient populations and healthcare practices. The small sample size of the study may not have been large enough to capture all potential risk factors or to detect subtle differences in outcomes between groups. Additionally, since the study lacks long-term follow-up data on patient outcomes beyond their ICU stay, we cannot provide data on the chronic effects of COVID-19 and renal injury. While the association between COVID-19 and AKI is well-documented, we cannot clearly differentiate correlation from causation. Therefore, it would benefit from a deeper analysis of how COVID-19 leads to renal injury beyond just statistical associations.

## Conclusions

COVID-19, primarily a respiratory disease, poses a significant risk of AKI or exacerbation of pre-existing renal conditions, especially among elderly males with comorbidities such as hypertension, coronary artery disease, chronic liver disease, CKD, malignancy, and irregular treatment for chronic diseases. When compared to controls, individuals with renal injury experienced short-duration fever and overall shorter ICU stays. Our study highlights the association of biomarkers such as CRP, serum ferritin, and IL-6 with renal injury in COVID-19 patients. None of the treatment modalities showed significance in preventing mortality, indicating that supportive treatment and renal replacement therapy in indicated cases were the mainstays against COVID-19 with renal injury. Emphasizing the monitoring of kidney function, even in patients with mild respiratory symptoms, and giving particular attention to altered kidney function after admission in clinical practice are crucial. Early detection and treatment of renal abnormalities, including adequate hemodynamic support and avoidance of nephrotoxic drugs, may help improve the vital prognosis of COVID-19. Long-term follow-up is essential to understand the impact of resolved or unresolved AKI on the development of CKD and other post-COVID-19 complications.
